# Synthesis and Swelling Behavior of pH-Sensitive Semi-IPN Superabsorbent Hydrogels Based on Poly(acrylic acid) Reinforced with Cellulose Nanocrystals

**DOI:** 10.3390/nano7110399

**Published:** 2017-11-20

**Authors:** Lim Sze Lim, Noor Afizah Rosli, Ishak Ahmad, Azwan Mat Lazim, Mohd Cairul Iqbal Mohd Amin

**Affiliations:** 1 Polymer Research Centre (PORCE), School of Chemical Sciences and Food Technology, Faculty of Science and Technology, Universiti Kebangsaan Malaysia, Bangi Selangor 43600, Malaysia; modulus.s91@gmail.com (L.S.L.); noorafizahr@gmail.com (N.A.R.); azwanlazim@ukm.edu.my (A.M.L.); 2 Faculty of Pharmacy, University Kebangsaan Malaysia, Jalan Raja Muda Abdul Aziz, Kuala Lumpur 50300, Malaysia; mciamin@ukm.edu.my

**Keywords:** acrylic acid, chemical cross-linking, cellulose nanocrystals

## Abstract

pH-sensitive poly(acrylic acid) (PAA) hydrogel reinforced with cellulose nanocrystals (CNC) was prepared. Acrylic acid (AA) was subjected to chemical cross-linking using the cross-linking agent MBA (*N*,*N*-methylenebisacrylamide) with CNC entrapped in the PAA matrix. The quantity of CNC was varied between 0, 5, 10, 15, 20, and 25 wt %. X-ray diffraction (XRD) data showed an increase in crystallinity with the addition of CNC, while rheology tests demonstrated a significant increase in the storage modulus of the hydrogel with an increase in CNC content. It was found that the hydrogel reached maximum swelling at pH 7. The potential of the resulting hydrogels to act as drug carriers was then evaluated by means of the drug encapsulation efficiency test using theophylline as a model drug. It was observed that 15% CNC/PAA hydrogel showed the potential to be used as drug carrier system.

## 1. Introduction

A hydrogel can be defined as a three-dimensional network of a hydrophilic polymer that can absorb and retain large amounts of water within its network [1]. In order to maintain the structures of hydrogel networks in aqueous solution and prevent their dissolution, the development of either a physical or chemical cross-linking method was necessary [2]. To date, hydrogels have been developed for application in a range of fields, including personal hygiene products, and in biomedical, pharmaceutical, and mechanical engineering [3,4,5]. Particular attention has been given to “smart” hydrogels such as poly(acrylic acid) (PAA) and poly(acrylamide). The “smart” hydrogels are known for their response to the stimuli-sensitive changes in environmental conditions. For this reason, the “smart” hydrogels have been extensively studied [6]. These two hydrogels display sensitivity to changes in temperature and pH, which are both important factors for the application of hydrogels in medical applications [7]. 

Cellulose can be considered the most abundant naturally occurring polymer [8]. Over the past decade, studies on the extraction and modification of cellulose nanocrystals (CNCs) have been carried out to take advantage of the potential of CNCs to be used as raw materials in a range of applications [9,10]. CNCs were chosen as a biomaterial for hydrogel preparation due to their interesting characteristics, including favorable mechanical properties, low density, hydrophilicity, biodegradability, and high biocompatibility [11]. 

In terms of medicinal applications of hydrogels, drug carrier systems have been found to be advantageous in terms of increasing the residence time of drugs in patients, reducing the frequency of doses, reducing toxic effects of the drugs, and improving patient compliance [12]. However, drug carrier systems have a number of weaknesses that first need to be overcome, including their tendency to burst due to high swelling rates, and the low mechanical properties of the hydrogels. In addition, the low tolerance of hydrogels to acidic conditions in the stomach may cause undesired drug degradation [13]. Recently, some strategies have been explored for developing a new drug delivery system, where the covalent linkage of a given drug on a suitable matrix, as well as the possibility of inducing stimulus-responsive behaviors, are important tasks [14].

We therefore aim to prepare a pH-responsive hydrogel in the form of a semi-interpenetrating polymer network (IPN) by cross-linking acrylic acid monomers in a CNC suspension. Combining the advantageous characteristics of both polymers, the resulting hydrogels are expected to have a high degree of crystallinity and favorable mechanical properties. In addition, reducing the quantity of chemical cross-linking agent used, along with fine-tuning of hydrogel swelling, drug loading, and drug release behavior should be possible with the addition of CNC. Furthermore, the fact that cellulose cannot be digested by the human body due to the lack of the cellulase enzyme allows the resulting hydrogel to have high resistance to the stomach’s acidic environment.

## 2. Materials and Methods

### 2.1. Materials

Sodium chlorite (NaClO_2_), sodium hydroxide (NaOH), sulphuric acid (H_2_SO_4_) and glacial acetic acid (99%) were used for the isolation of CNC. Acrylic acid (99%) was purchased from Sigma Aldrich (Darmstadt, Germany). Laboratory-grade *N*,*N*′-methylenebis(acrylamide) (MBA, 99%), ammonium persulphate (APS, 99%), and theophylline (>99%) were purchased from Sigma-Aldrich (Darmstadt, Germany) for use as the cross-linker, initiator, and drug, respectively.

### 2.2. Preparation of PAA/CNC Hydrogels

Kenaf fibers were firstly ground and treated with 4% NaOH solution for 3 h at 90 °C under reflux condition. The purpose of alkali treatment is to remove hemicellulose content from the fibers. A bleaching treatment was then carried out using 1.7 *w*/*v* % NaClO_2_ at pH 4.5 and 100 °C for 4 h under reflux condition. The bleached fibers were then subjected to hydrolysis using concentrated H_2_SO_4_ (65%) to remove amorphous region. The reaction was carried out at 45 °C under constant stirring for 45 min. The hydrolysis reaction was terminated after 45 min by placing the reaction flask in an ice bath. The suspension was then centrifuged at 4500 rpm. It was then placed into dialysis tube and dialyzed against distilled water until pH was neutral. The resultant suspension was then freeze-dried to obtain CNC powder. The characteristics of CNC is summarized in Table 1.

The hydrogels were prepared by cross-linking an AA monomer in a suspension of CNC, using APS as the initiator and MBA as the cross-linker. A typical experimental process for the preparation of a 5 wt % sample is as follows. A mixture of water (10 mL), AA (4.75 g), and CNC (0.25 g) was prepared and homogenized. A solution of MBA (3%, 10 mL) was then added, and the resulting mixture heated with stirring for 20 min. After this time, a solution of APS (2%, 5 mL) was added at 50 °C and the resulting homogenous mixture was stirred and heated until a temperature of 60 °C was reached. 

The mixture was then cast onto a pre-heated petri dish and allowed to cool. The resulting hydrogels were immersed in distilled water for 24 h to remove any unreacted reagents from the hydrogel. The leeched hydrogels were then dried to constant weight in an oven at 70 °C. The exact compositions of the hydrogels are given in Table 2.

### 2.3. Characterizations

The prepared hydrogel samples were subjected to FTIR (Perkin Elmer, Spectrum GX, Hopkinton, MA, USA) in order to verify the successful cross-linking of the AA monomers, and the formation of a semi-IPN hydrogel following the addition of CNC. The spectra were recorded between 400 and 4000 cm^−1^. 

XRD analysis was conducted using a D8-Advance (Bruker AXS GmbH, Oestliche Rheinbrueckenstr, Karlsruhe, Germany) to investigate the changes in the hydrogel crystallinity following CNC addition. The swollen hydrogel samples were first freeze-dried, before being ground into a fine powder. CuKα (*λ* = 0.1539 nm) was used as a radiation source and was directed towards the sample with a 2*θ* angle of 5–80°, generated at 40 kV and 30 mA. 

The equilibrium swelling ratios of the hydrogels in response to pH were studied using various buffer solutions ranging from pH 3 to pH 11. The dried hydrogels were cut into small pellets, immersed in the desired buffer solution (50 mL), and allowed to soak for 48 h. At predetermined time intervals, the immersed samples were removed from the buffer solution and weighed after gently wiping the surface of the hydrogel using filter paper. The equilibrium swelling ratio can be determined using the following equation:

(1) $$\mathrm{Equilibrium}\;\mathrm{swelling}\;\mathrm{ration}\;=\;\frac{W_{s}-W_{d}}{W_{d}}\times 100$$

where *W*_s_ and *W*_d_ represent the weight of the swollen hydrogel and the weight of the dry gel, respectively. The average value obtained from five tests was taken for each sample.

The rheological behavior of the swollen hydrogels was studied using a Physica MCR 301 rheometer (Anton Paar) parallel plate system (Petaling Jaya, Selangor, Malaysia). Strain sweep tests were first carried out in order to determine the linear viscoelastic ranges of the hydrogels where the storage modulus (G’) and loss modulus (G”) were independent of the strain amplitude. Frequency sweep tests were then carried out to study the viscoelastic behavior of the hydrogels. G’ and G” were investigated as a function of angular frequency (*ω*) from 0.1 to 100 rad/s at constant strain and temperature.

Scanning electron microscopy (SEM) micrographs of the PAA and PAA composite hydrogels were recorded on a Philips XL 30 scanning electron microscope (North Billerica, MA, USA) with an accelerating voltage of 20 kV. Samples for SEM were first cryo-fractured from liquid nitrogen and then dried for 36 h. The resulting fracture surface was coated with gold prior to the measurements, to avoid charging.

Theophylline, which is commonly used as a model drug [15], was loaded onto the hydrogel using a separation diffusion method. Dried disk-shaped hydrogel samples were immersed in the drug solution (10 mL, 5 mg/mL) for 24 h. The swollen hydrogels were removed from the drug solution and rinsed with distilled water (10 mL) to remove any drug solution remaining on the hydrogel surface. The hydrogels were then left to dry in an oven at 37 °C. The concentrations of the remaining drug solutions were determined using UV spectrophotometry (UV-1800, Shimadzu, Tokyo, Japan) at 272 nm. The drug loading efficiency (*EE*) for the hydrogel can be calculated according to the following equation:

(2) $$EE\;(\%)=(\frac{D_{L}}{D_{I}})\times 100$$

where $D_{L}$ is the amount of drug loaded onto the hydrogel, and *D*_I_ is the total amount of drug in the solution.

Drug release studies were performed by soaking the drug-loaded hydrogel in a buffer solution (100 mL) at pH 7.4 for 24 h. The temperature of the buffer solution was maintained at 37 °C with constant stirring at 50 rpm. Aliquots of the buffer solution (2 mL) were removed at predetermined time intervals and replaced with 2 mL of fresh buffer solution to maintain the total volume of the stock buffer solution. The drug concentrations in the samples of buffer solution were then determined using a UV-1800 spectrophotometer (Shimadzu, Tokyo, Japan) at 272 nm.

## 3. Results and Discussion

### 3.1. Fourier Transform Infrared (FTIR) Spectroscopy

**Figure 1:**
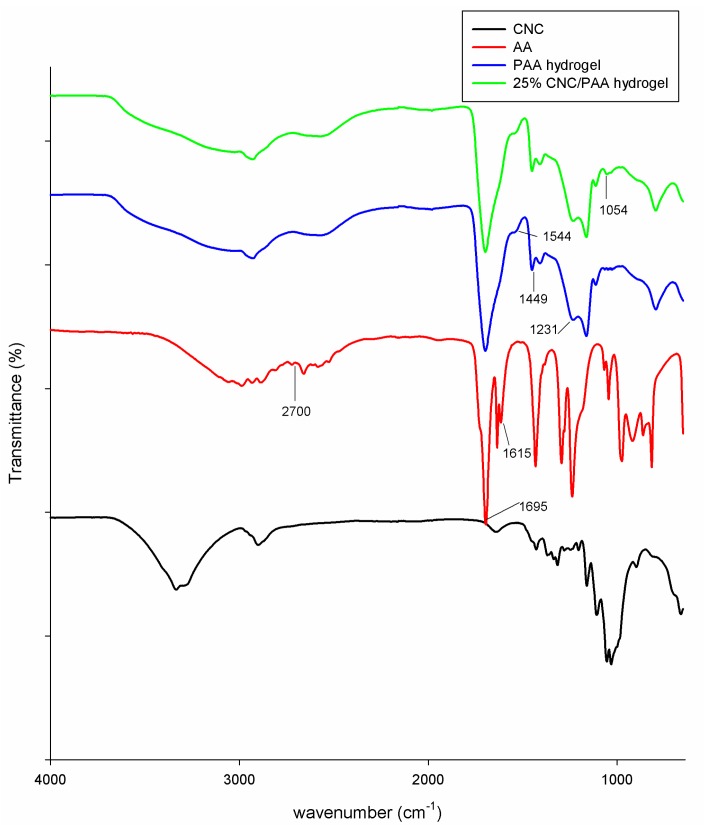
FTIR (Fourier Transform Infrared) spectra of CNC, AA, PAA hydrogel, and 25% CNC/PAA hydrogel.

Figure 1 shows the FTIR spectra for CNC, AA, PAA hydrogel, and 25% CNC/PAA hydrogel. In order to investigate the functional group changes following cross-linking and the addition of CNC, the AA spectrum was used for comparison. The AA spectrum showed distinctive peaks at approximately 1695 and 1615 cm^−1^, corresponding to the carboxylic C=O moiety and to C=C stretching, respectively. Broad bands between 2700 and 3200 cm^−1^ can be assigned to the hydroxyl groups [16,17]. 

In the case of the PAA hydrogel spectrum, the peak at 1449 cm^−1^ can be assigned to the CH_2_ bending vibration [18], while that at approximately 1231 cm^−1^ can be assigned to C–O stretching [19]. Comparison of the PAA hydrogel spectrum with that of AA shows that the peak at approximately 1615 cm^−1^, corresponding to C=C stretching had disappeared. This confirmed that the cross-linking reaction had been successful, as during cross-linking, MBA cross-linked with the C=C groups in PAA chains at random, resulting in the formation of C–C moieties. This was further confirmed by the appearance of a new peak at 1544 cm^−1^, which corresponds to C–C stretching [20]. 

**Figure 2:**
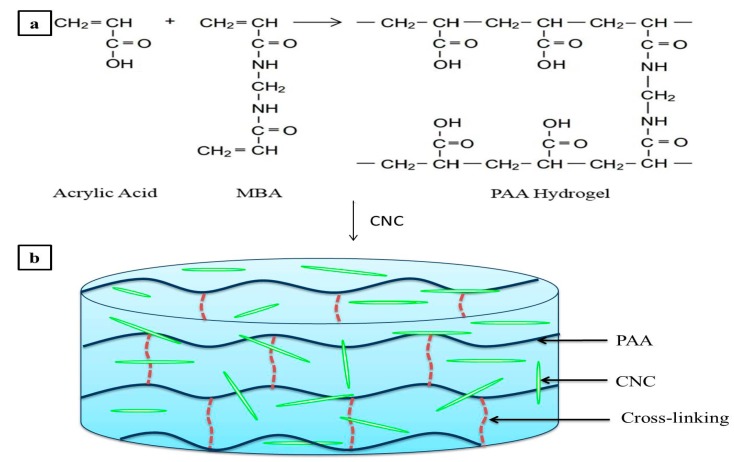
(**a**) Cross-linking of AA to give PAA; and (**b**) the proposed mechanism for the formation of the semi-IPN hydrogel following CNC addition.

Peaks from the FTIR spectra of both PAA and CNC were displayed in the spectrum of the PAA hydrogel reinforced with 25% CNC. However, due to both a low CNC concentration compared to PAA and a homogenous distribution of CNC in PAA, only the highest-intensity CNC peaks were observed in the spectrum of the semi-IPN hydrogel. For example, the peak at approximately 1054 cm^−1^, which can be assigned to the C–O groups on CNC, was observed, thus confirming that the hydrogels were in the semi-IPN form, with both materials maintaining their own distinctive properties. The mechanism of PAA cross-linking [21] and the proposed mechanism for the formation of semi-IPN hydrogels are illustrated in Figure 2.

### 3.2. X-ray Diffraction Analysis

**Figure 3:**
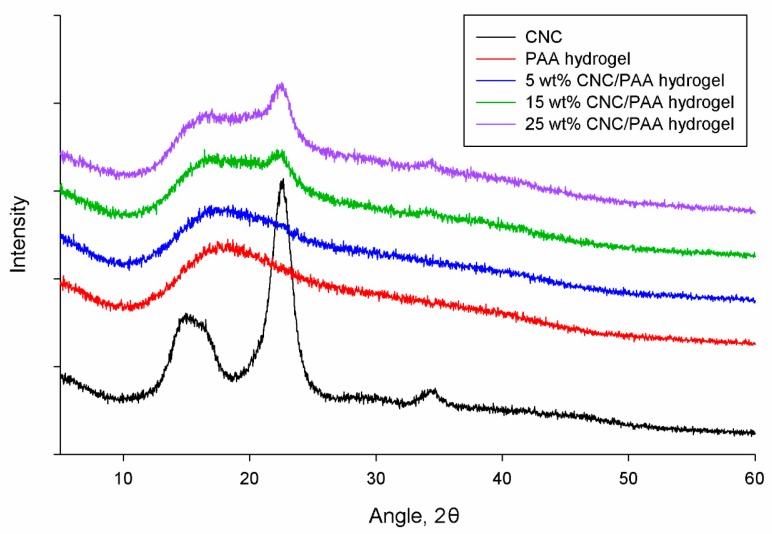
XRD diffractograms of CNC, PAA hydrogel, 5 wt % CNC/PAA hydrogel, 15 wt % CNC/PAA hydrogel, and 25 wt % CNC/PAA hydrogel.

The XRD diffractograms recorded from 2*θ* = 5° to 80° for CNC, PAA hydrogel, 5 wt % CNC/PAA hydrogel, 15 wt % CNC/PAA hydrogel, and 25 wt % CNC/PAA hydrogel are shown in Figure 3. A typical cellulose I diffraction pattern with well-defined crystalline peaks at 2*θ* = 16°, 23° and 34° can be observed in the diffractogram of CNC [22]. CNC has a high degree of crystallinity, due to acid hydrolysis cleaving away the amorphous region of CNC and leaving the crystalline domain intact. Due to the orderly hydrogen-bonding arrangement between cellulose molecules, it is more difficult for acid to penetrate and hydrolyze these crystalline regions [23,24]. 

It was also found that no peaks corresponding to CNC were observed in the 5 wt % CNC/PAA hydrogel due to the low CNC concentration present in this hydrogel. However, in the 15 wt % CNC/PAA hydrogel diffractogram, CNC peaks at 2*θ* = 16° and 23° began to appear, and became shaper and clearer in the diffractogram of 25 wt % CNC/PAA. With the incorporation of these highly crystalline materials, the resulting hydrogels are expected to be stiffer and able to withstand higher shear stress.

### 3.3. Swelling Studies

**Figure 4:**
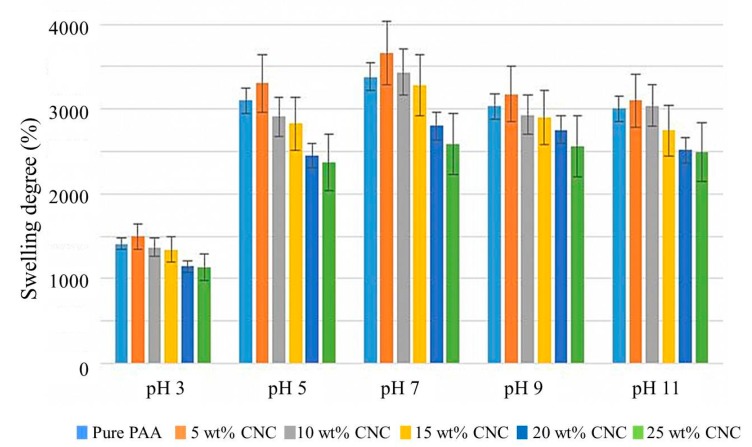
Swelling ratios of hydrogels with different CNC compositions, at pH 3, pH 5, pH 7, pH 9, and pH 11.

The equilibrium swelling ratios of hydrogels with different CNC compositions are illustrated in Figure 4. For all pH values tested, the 5 wt % CNC/PAA hydrogel was found to display the highest equilibrium swelling ratio, followed closely by the pure PAA hydrogel. It was found that the incorporation of more than 5 wt % CNC tended to decrease the swelling ratio. The increase in equilibrium swelling ratio observed for the 5 wt % CNC/PAA hydrogel is likely due to an increase in number of hydrophilic hydroxyl groups [25] facilitating water sorption into the hydrogel. However, at greater CNC contents (>5 wt %), the CNC fills the voids between the polymer chains, thus producing a tougher and more rigid hydrogel structure. The increase in crystallinity also creates a barrier that prevents water molecules penetrating the hydrogel network, and thus a decrease in swelling ratio is observed.

PAA could be considered a polyelectrolyte polymer, as it possesses a number of ionizable pendant acidic groups. Low swelling ratios observed at pH 3 can be assigned to the unfavorable condition for the COOH groups in PAA to ionize, whose pKa is 4.25 [26]. However, the swelling ratio increases drastically at pH 5 until reaching a maximum at pH 7, before the swelling ratio decreases at higher pH. The sudden rise in equilibrium swelling ratio observed at pH 5 was likely due to the ionization of the COOH groups of PAA into negatively charged COO^−^ moieties. The resulting repulsive electrostatic forces generated from the COO^−^ groups caused the hydrogel network to expand, and thus increased the swelling ratio. 

### 3.4. Rheology

**Figure 5:**
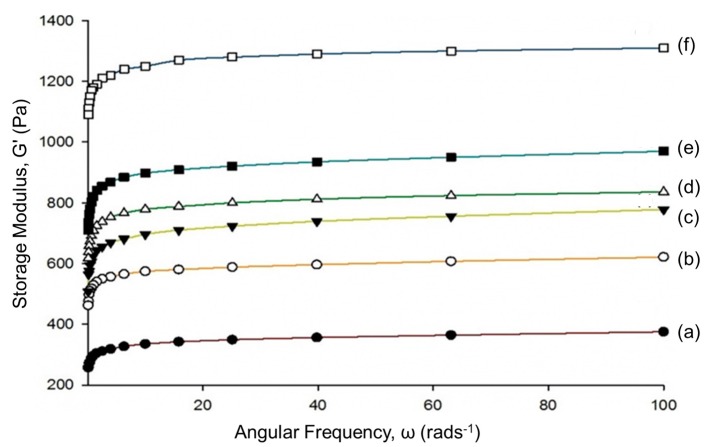
Storage moduli, G’ of (**a**) PAA hydrogel; (**b**) 5 wt % CNC/PAA hydrogel; (**c**) 10 wt % CNC/PAA hydrogel; (**d**) 15 wt % CNC/PAA hydrogel; (**e**) 20 wt % CNC/PAA hydrogel; and (**f**) 25 wt % CNC/PAA hydrogel.

Figure 5 shows the storage moduli of the neat and semi-IPN hydrogels as a function of shear frequency. The rheological properties of a viscoelastic material are usually characterized according to its storage modulus (G’) and loss modulus (G”), where G’ is a measurement of the elasticity or solid-like behavior of a material, and G” is a measurement of the viscosity or liquid-like behavior of a material. In the study reported herein, we chose to focus on the G’ of the hydrogels, as the incorporation of CNC was expected to increase the dynamic mechanical properties of the hydrogel (i.e., the hydrogel was expected to be more solid-like). 

As can clearly be seen in Figure 5, the G’ values of hydrogels were found to increase with increasing CNC content, with the 25 wt % CNC/PAA hydrogels showing a maximum G’ value of 1310 Pa at 100 s^−1^. This demonstrates that the hydrogels became tougher and more rigid with the addition of CNC. These results are consistent with the XRD findings, where it was found that the incorporation of CNC increased the overall crystallinity of the hydrogels. This can be explained in terms of CNC strengthening the hydrogel in a similar manner to other nano-sized fillers, such as carbon nanotubes and nano clay [27]. In addition, the homogenous dispersion of CNC in the hydrogels led to an increase in the stress transfer from the polymer chains to CNC, thus preventing the formation of micro cracks in the hydrogel. Furthermore, due to the hydrophilic nature of both PAA and CNC, the formation of excellent interfaces between the two also contributed to the formation of a strong hydrogel [28].

### 3.5. Morphological Analysis

**Figure 6:**
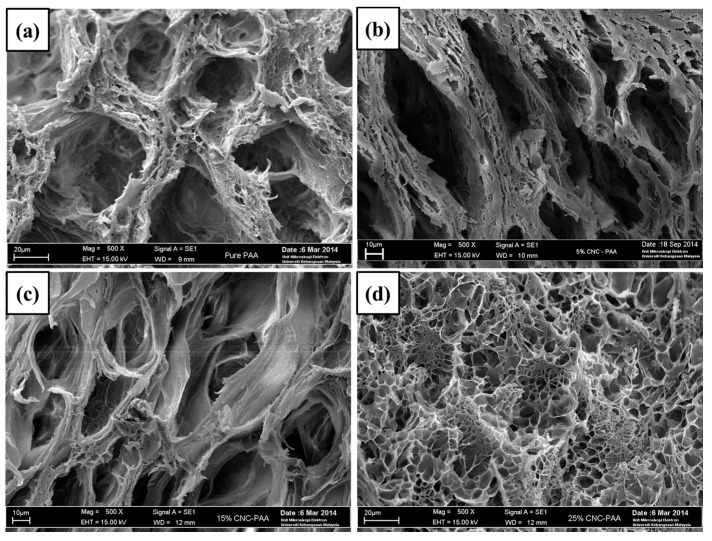
SEM micrographs of cross-section of (**a**) PAA hydrogel; (**b**) 5 wt % CNC/PAA hydrogel; (**c**) 15 wt % CNC/PAA hydrogel; and (**d**) 25 wt % CNC/PAA hydrogel.

Figure 6 shows the cross-section morphology of hydrogels with varying CNC contents. The purpose of the test is to investigate the effect of CNCs loading on the pores size of the hydrogels. Furthermore, the morphology study of the hydrogels cross-section helps to identify the relationship between the pores size with the degree of crystallinity, mechanical properties and swelling ratio of the hydrogels. From the micrographs, all of the hydrogels showed a very porous and sponge-like structure. It is interesting to note that the changes in pores size were coherent with the swelling test results, in which 5 wt % CNC/PAA hydrogel showed the largest pore size, followed by the PAA hydrogel. The higher the swelling ratio (high water content), the greater the size of the hydrogel pores obtained after the freeze-drying process, and vice versa.

The pore sizes of both 15 wt % CNC/PAA and 25 wt % CNC/PAA hydrogel were found to be smaller than that of 5 wt % CNC/PAA hydrogels. The same observation has been reported previously, where the size of the hydrogel pores decreased with the addition of nano-size chitosan fibers [28]. Moreover, it can be clearly seen that the pore distribution in 25 wt % CNC/PAA hydrogel was more uniform than that in the other hydrogels, which indicates a more ordered polymer network. This will eventually prevent uneven pore production during the freeze-drying process. Normally, this ordered polymer network would also allow uniform pressure distribution within the polymer matrix, thereby increasing the mechanical properties of the hydrogel, as shown in Figure 5.

### 3.6. Drug Loading

**Figure 7:**
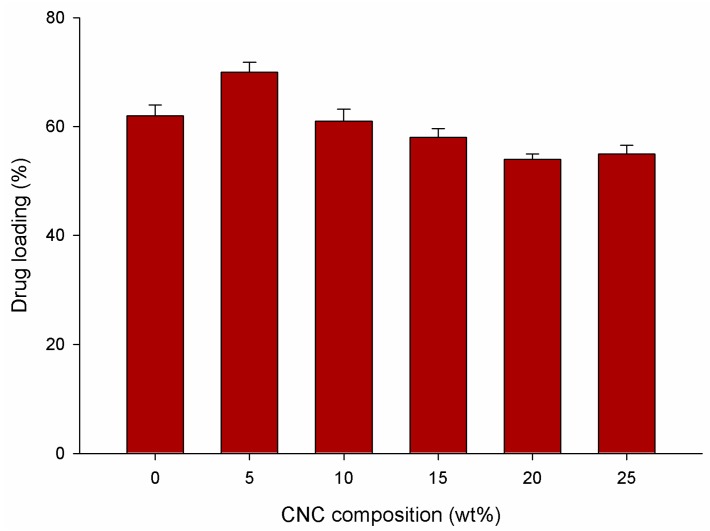
Drug loading (%) on the hydrogel at varying CNC composition.

The degree of drug loading (%) on the hydrogels at a range of CNC compositions was then investigated, as it is an important factor in the evaluation of hydrogels as potential drug carriers. An ideal drug carrier should be capable of trapping large quantities of the drug molecule inside its network to avoid waste, and therefore reduce the number of doses. As can be seen in Figure 7, the total drug loading trend was in agreement with the swelling test results, with the 5 wt % CNC/PAA hydrogel followed by the pure PAA hydrogel achieving the highest drug loading percentage. This could be a result of possible H-bonding between the hydroxyl group of hydrogels and amine groups of the theophylline. 

As before, a further increase in CNC content tended to lead to a drop in drug loading. These results demonstrate that the drug loading is directly related to the equilibrium swelling ratio, as the greater the quantity of a drug solution that a hydrogel can retain in its network, the greater the resulting drug loading of the hydrogel. Furthermore, the drug solution was loaded into the hydrogels through the diffusion method, meaning that a higher swelling ratio would lead to a higher drug loading efficiency. The content of the drug loading can be controlled by varying the weight of the hydrogel. In this study, 34.7 mg of the drug was able to be loaded in 110 mg of 5 wt % CNC/PAA hydrogel, as shown along with others in Table 3.

### 3.7. Drug Release

**Figure 8:**
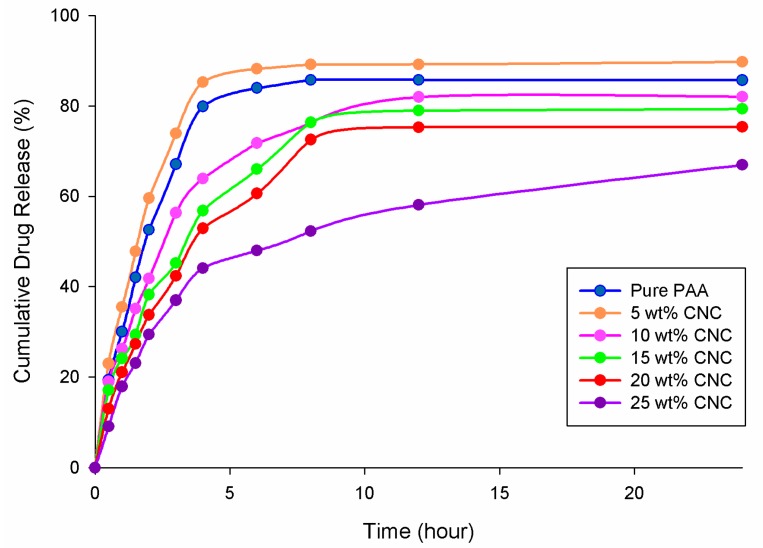
Cumulative drug release of hydrogels with varying CNC compositions.

Figure 8 shows the cumulative drug release profiles of hydrogels with varying CNC content. Upon examination of the plot, it can be seen that all hydrogels show a drug release pattern that can be divided into two phases: (1) A sudden burst; and (2) Continuous drug release. The burst effect observed in the first hour can be explained as the release of the drug present on the surface of the hydrogels [29]. Although the hydrogel samples were rinsed with distilled water after drug loading, there still appeared to be traces of drug remaining on the surface. It was found that 5 wt % CNC/PAA showed the most significant burst effect, followed by the PAA hydrogel, with an increase in CNC content reducing the degree of the burst effect. The enhanced burst effect observed with the 5 wt % CNC/PAA hydrogel was attributed to the hydrogel achieving swelling equilibrium faster than the other hydrogels, thus resulting in the drug molecules diffusing more quickly into the surrounding buffer solution.

At the beginning of the second phase, the rate of drug release from the hydrogel is fast, but decreases gradually over time. This occurs due to a low drug concentration in the buffer at the beginning of this phase, and so the concentration gradient acts as a driving force for drug release. As the drug concentration in the buffer solution increases, the concentration gradient decreases and thus the rate of drug release drops [30]. Although the 5 wt % CNC/PAA hydrogel showed the highest cumulative release after 24 h, it is not suitable for use as a drug carrier, as the rate of drug release was too high. The same conclusion can also be made for both the PAA hydrogel and the 10 wt % CNC/PAA hydrogel. In contrast, the rates of drug release from the 20 wt % CNC/PAA hydrogel and 25 wt % CNC/PAA were too slow, and neither reached equilibrium after 24 h. 

The mathematical treatment of experimental data was carried out to understand the kinetic mechanism that directs the systems. To quantify and materialize the amount of drug released by Fickian diffusion and polymer relaxation, the Peppas-Sahlin model was chosen. The drug release data is treated based on the Peppas-Sahlin equation [31].

(3) $$\frac{M_{t}}{M_{\infty }}{=K}_{1}{}^{n}{+K}_{2}{}^{2n}$$

where *M_t_*/*M*_∞_ is the fraction of the drug release at a time, *t* and n is diffusional exponent. K_1_ and K_2_ are kinetic constant. The equation is the sum of two terms of Fickian mechanism and the relaxation mechanism of polymeric chains.

The results of applying this model to the experimental data obtained using the hydrogel with different CNC composition are shown in Table 4. It is noted that all of the regression coefficients of the hydrogels are higher than 0.90, which is high enough to evaluate the release data according to Peppas-Sahlin equation. The higher values of the diffusion constant, K_1_, compared with the relaxation constant, K_2_, combined with the high solubility of the drug, suggest the prevalence of swelling mechanism for drug release over erosion mechanism [31].

In the Peppas-Sahlin model, drug release follows the Fickian-type diffusion mechanism when *n* = 0.45. Meanwhile, when the values of n are higher than 0.45 but less than 1, an abnormal diffusion or non-Fickian diffusion occurs. Lastly, when *n* = 1, the kinetics of the release system is zero order (transport Case II) [32]. From Table 4, it can be clearly seen that the n values obtained from this model are between 0.45 and 1, thus following an abnormal or non-Fickian diffusion. This means the diffusion rate depends on the drug concentration gradient. 

## 4. Conclusions

In this study, pH-responsive semi-IPN hydrogels were synthesized by cross-linking AA monomers in a suspension of CNC using MBA as the cross-linking agent. The resulting hydrogels showed an increase in crystallinity with the addition of CNC. In addition, the storage modulus of the hydrogel increased significantly with incorporation of CNC, proving its effectiveness for improving the mechanical properties of the hydrogel. Swelling tests showed that the hydrogels achieved optimum swelling at pH 7, with the 5 wt % CNC/PAA hydrogel displaying the highest swelling ratio. This study clearly demonstrated that the hydrogel has the potential to be used as a drug carrier based on drug loading and drug release performance.

## Figures and Tables

**Table 1:** The properties of CNC from kenaf fiber.

Sample	Equivalent Diameter (nm)	Equivalent Length (nm)	Aspect Ratio
CNC	4.5 ± 1.5	228 ± 38	32–89

**Table 2:** Hydrogel composition.

Sample	CNC (g)	AA (g)	MBA (g)	APS (g)
Neat AA	-	5.00	0.15	0.1
5 wt %	0.25	4.75	0.1425	0.095
10 wt %	0.5	4.5	0.135	0.09
15 wt %	0.75	4.25	0.1275	0.085
20 wt %	1	4	0.12	0.08
25 wt %	1.25	3.75	0.1125	0.075

**Table 3:** Data of drug loading by weight.

Hydrogel	Weight of Hydrogel (mg)	Weight of Drug Loading (mg)
PAA	99 ± 20	31 ± 1
5 wt % CNC/PAA	110 ± 18	34.7 ± 0.9
10 wt % CNC/PAA	110 ± 22	30.5 ± 1.1
15 wt % CNC/PAA	110 ± 16	29 ± 0.8
20 wt % CNC/PAA	110 ± 10	27 ± 0.5
25 wt % CNC/PAA	110 ± 16	27.5 ± 0.8

**Table 4:** Fitting parameters obtained from the Peppas-Sahlin equation.

CNC Composition (wt %)	Regression Coefficient (*R*^2^)	Kinetic Constant (K_1_)	Kinetic Constant (K_2_)	*n*
0	0.997	32.10	−0.55	0.72
5	0.998	37.19	−0.13	0.69
10	0.993	29.46	−0.73	0.61
15	0.993	25.79	−0.49	0.58
20	0.994	20.32	−0.30	0.64
25	0.929	12.83	0.35	0.53
